# Eltrombopag with or without Tacrolimus for relapsed/refractory acquired aplastic anaemia: a prospective randomized trial

**DOI:** 10.1038/s41408-023-00921-8

**Published:** 2023-09-19

**Authors:** Jiang Ji, Ziqi Wan, Jing Ruan, Yuan Yang, Qinglin Hu, Zesong Chen, Chen Yang, Miao Chen, Bing Han

**Affiliations:** Peking Union Medical College Hospital (PUMCH), Chinese Academy of Medical Sciences and Peking Union Medical College, Beijing, China

**Keywords:** Medical research, Anaemia

## Abstract

This trial compared eltrombopag (EPAG)+tacrolimus and EPAG monotherapy in patients with refractory/relapsed acquired aplastic anaemia (AA). Patients with refractory/relapsed AA were randomly assigned to receive either EPAG+tacrolimus or EPAG monotherapy at a ratio of 2:1. Patient response, safety, clonal evolution and survival were compared. In total, 114 patients were included in the analysis, with 76 patients receiving EPAG+tacrolimus and 38 receiving EPAG only. With a median follow-up of 18 (6–24) months, the overall response rate (ORR) for patients treated with EPAG+tacrolimus and EPAG alone was 38.2% *vs*. 31.6% (*P* = 0.490) at the 3^rd^ month, 61.8% *vs*. 39.5% (*P* = 0.024) at the 6^th^ month, 64.5% *vs*. 47.1% (*P* = 0.097) at the 12^th^ month, and 60.5% *vs*. 34.2% (*P* = 0.008) at the last follow-up. The rate of each adverse event, overall survival curves (*P* = 0.635) and clonal evolution rate (*P* = 1.000) were comparable between the groups. A post hoc subgroup analysis showed that EPAG+tacrolimus could have advantage over EPAG monotherapy in terms of the ORR at the 6^th^ month (*P* = 0.030)/last follow-up (*P* = 0.013) and the cumulative relapse-free survival (RFS) curves (*P* = 0.048) in patients <60 years old.

## Introduction

Acquired aplastic anaemia (AA) is a mostly immune-mediated marrow failure syndrome [1, 2]. It is characterized with peripheral pancytopenia and hypocellular bone marrow, and could be life-threatening. Immunosuppressive therapy (IST) with antithymocyte globulin (ATG) and cyclosporine A (CsA) [3] or CsA alone is the first-line therapy for AA patients who are not transplant candidates [4, 5]. The response rate of severe AA (SAA) patients to ATG+CsA is 60%-80% [6–8]. In addition, approximately 10–30% of SAA patients treated with IST relapsed [8, 9].

EPAG, a thrombopoietin receptor agonist (TPO-RA), was reported to be efficient for treating AA. The overall response rate (ORR) to EPAG monotherapy was 50%-80% in treatment-naïve moderate AA patients [5], and 35%-60% in refractory/relapsed AA patients [10, 11]. In addition, Townsley et al. [12] found that EPAG combined with IST can improve the ORR of untreated SAA patients to 94%, and a phase-3 trial focused on SAA patients reported a higher complete response rate (CRR) to EPAG + IST therapy than to IST as first-line therapy [13]. However, little research reported the efficacy of EPAG+immunosuppressive therapy on refractory/relapsed AA patients, and no study has compared the efficacy of EPAG+immunosuppressive therapy and EPAG monotherapy in refractory/relapsed AA patients.

Here we conducted a randomized clinical trial for patients with refractory/relapsed AA; set two therapy groups (namely the EPAG+tacrolimus and EPAG monotherapy groups), and analysed patient response, safety, clonal evolution, and survival of both groups. We further analysed factors that could possibly influence the response rate or relapse rate associated with each therapy. We used tacrolimus as the alternative for CsA because tacrolimus has a more favorable toxicity profile and may have advantage for patients who were refractory to CsA.

## Subjects and methods

### Patients

From May 2020 to November 2021, patients with relapsed/refractory acquired AA who came to Peking Union Medical College Hospital (PUMCH) were enrolled according to the following criteria: (1) age ≥14 years old; (2) established diagnosis of acquired AA [2] after a complete workup, including bone marrow biopsy, cytogenetic examination, autoimmune antibody testing, virus infection history and screening for myelodysplastic syndrome (MDS)-associated genetic mutations; (3) classification of SAA or non-severe AA (NSAA) but with at least one of the following: (A) an absolute neutrophil count <0.5 × 10^9^/L, (B) a haemoglobin level <90 g/L, and (C) a platelet count <30 × 10^9^/L before enrolment [10]; and (4) previous standard IST, namely CsA + ATG or CsA alone for at least 6 months, but relapsed or failed to respond. Patients meeting any of the following criteria were excluded: (1) specific tests indicating congenital AA (e.g. fanconi anaemia and congenital dyskeratosis. Chromosome breakage test, single cell gel electrophoresis and length of telomere were conducted in patients <20 years old. Germline next-generation sequencing was performed to assess congenital AA in patients with abnormal functional testing or clinical features suspicious for an inherited bone marrow failure syndrome); (2) a transplantation history; (3) a paroxysmal nocturnal haemoglobinuria (PNH) granulocyte clone size ≥50% (measured by peripheral blood flow cytometry for fluorescently labelled modified aerolysin (FLAER)); (4) concomitant clonal haematologic disorders; (5) concomitant malignancies; (6) pregnancy or lactation; (7) allergy to EPAG and/or tacrolimus; (8) ALT or AST ≥ 3 times the upper limit of normal range; (9) serum creatinine ≥ 1.5 times the upper limit of normal range; (10) infection not adequately responding to appropriate therapy; and (11) prior treatment with EPAG or other TPO-RAs. This study was approved by the ethics committee of PUMCH. All the eligible patients were fully informed of the study protocol and signed written informed consent forms.

### Study design

This was a single-centre, prospective, randomized clinical trial registered at www.clinicaltrial.gov (NCT04403321). After the consent form was signed, the enrolled patients were randomly assigned into EPAG+tacrolimus group or EPAG monotherapy group at the ratio of 2:1 to increase the amount of information obtained on the new intervention. The randomization was balanced for disease severity (SAA or NSAA) between the groups. EPAG was started at 25 mg/day for 2 weeks, and was increased every 2 weeks until the best response was achieved or the dose reached 150 mg/day. When the best response was achieved, the dose of EPAG was maintained for 3 months and then tapered gradually (25 mg/day every 3 months); otherwise, patients were treated with EPAG for at least 6 months before the last evaluation for response. Tacrolimus was started at 1 mg b.i.d., and the patient’s plasma trough concentration was tested every 4 weeks for the first 6 months and every 8 weeks after that, so the dose of tacrolimus could be adjusted to maintain a plasma trough concentration of 4–10 ng/mL. Once the patient responded, tacrolimus was continued for at least 1.5 years. Data on patient baseline characteristics (including sex, age, the severity of AA [2], and the regimen and dosage of previous therapies, etc.) were collected. A complete workup was repeated for patients to confirm the diagnosis of AA before randomization. Baseline laboratory examinations including complete blood counts, liver and kidney function tests, PNH clone size determination, bone marrow smear and biopsy, chromosome and gene examinations if possible were performed and the results were recorded. Regular follow-up findings, including patients’ symptoms, signs and laboratory examinations, the dosage of EPAG, response, adverse events (AE) and final outcome were recorded and documented.

### Clinical evaluation and measures

Haematologic response was classified as complete response (CR), partial response (PR), overall response (OR = CR + PR) and no response (NR), and the response in each blood cell lineage was defined according to published literature [2, 11]. AEs attributed to either EPAG or tacrolimus were evaluated via Common Terminology Criteria for Adverse Events (CTCAE) version 3 of the National Cancer Institute [11]. The primary outcome measure was the ORR at the 6^th^ month of follow-up, and the secondary outcome measures were the ORR at the 3^rd^ month of follow-up. Haematologic response and potential AEs during EPAG treatment were evaluated at the 3^rd^, 6^th^, and 12^th^ months and at the last follow-up. Relapse was defined as a substantial or progressive decline in the count of at least one blood lineage of blood counts that required the reinitiation or augmentation of treatment [11]. A post-hoc relapse-free survival (RFS) and age subgroup analysis was conducted. Patients <60 years old and ≥60 years old were analysed separately to evaluate CRR, ORR, AE rate, clonal evolution and RFS.

### Statistical analysis

The estimated sample size was calculated based on the ORR according to historical studies [14]; from this calculation, the estimated sample size of the EPAG monotherapy group was 38 patients. This sample size would provide the trial with 80% power to reject the null hypothesis at a 5% significance level [13]. According to our clinical experience and previous studies [15], the course of EPAG for AA patients should be no less than 6 months to allow patients reach an ideal response; in addition, during our preliminary analysis, the censoring of patients who dropped out within 6 months of enrolment did not alter the results significantly (numbers of censored patients in both groups were illustrated in Supplementary Fig. 1), so these patients were not included or replaced in our final analysis. Descriptive statistics are presented as frequencies. Continuous variables are expressed as the median and range, or the mean ± SEM. Quantitative data were compared by a two-tailed t test or Mann-Whitney U test, while categorical data were compared using the chi-square test or Fisher’s exact test. For the analysis of response or relapse predictors, variables that were considered potentially related to response or relapse were included in the logistic regression analysis. Kaplan‒Meier curves were used for cumulative RFS and overall survival description and were compared by the log-rank test. A two-sided *P* < 0.05 was considered statistically significant. SPSS Statistics (version 25; IBM, Armonk, NY, USA) and GraphPad Prism software (version 5.00; GraphPad Software, San Diego, CA, USA) were used to perform the statistical tests.

## Results

### Patient baseline characteristics

A diagram of screening and enrolment details was depicted in Supplementary Fig. 1. Ultimately, 114 patients were included in the analysis, with 96 (84.2%) classified as NSAA patients and 18 (15.8%) classified as SAA patients. Among them, 59 (51.8%) patients were refractory to previous IST treatment, and the others were relapsed patients. Seventy-six patients received EPAG+tacrolimus and 38 received EPAG only. No significant difference was found in the baseline characteristics of patients between the EPAG+tacrolimus and EPAG monotherapy groups, including the SAA/NSAA ratio, previous ATG/ATG+CsA treatment ratio, time to previous IST treatment, patient baseline data before EPAG like laboratory examinations, PNH clone presence proportion, and cytogenetic characteristics (Table 1).

**Table 1:** Patient baseline characteristics.

Characteristics	EPAG+tacrolimus *N* = 76	EPAG *N* = 38	*P-*value
Age at EPAG initiation/years, median (range)	45.5 (14–83)	47 (14–79)	0.257
Age < 60 years old/*n* (%)	60 (78.9%)	25 (65.8%)	0.128
Male/*n* (%)	27 (35.5%)	19 (50.0%)	0.138
Severity of AA at EPAG initiation			
NSAA/*n* (%)	65 (85.5%)	31 (81.6%)	0.586
SAA/*n* (%)	11 (14.5%)	7 (18.4%)	
Disease status			
Relapsed/*n* (%)	36 (47.4%)	19 (50.0%)	0.791
Refractory/*n* (%)	40 (52.6%)	19 (50.0%)	
Previous treatment			
ATG + CsA/*n* (%)	11 (14.5%)	7 (18.4%)	0.586
CsA/*n* (%)	65 (85.5%)	31 (81.6%)	
Time to previous IST treatment/months, median (range)	29.5 (6-480)	62.5 (6-468)	0.193
ANC/×10^9^/L, median (range)	1.50 (0.18-7.45)	1.18 (0.61-4.45)	0.961
Platelet count/×10^9^/L, median (range)	12.5 (1-261)	15.5 (1–26)	0.786
Haemoglobin/(g/L), median (range)	81 (24-159)	69 (30-167)	0.248
Reticulocyte count/×10^9^/L, median (range)	56.6 (6.6-230.5)	51.0 (16.6-220.6)	0.624
Cr/(μmol/mL), median (range)	80 (38-168)	88 (40-178)	0.569
ALT/U/L, median (range)	14.5 (5-118)	18 (8–32)	0.413
Ferritin/ng/ml, median (range)	491 (10-4420)	1162 (12-9135)	0.234
PNH clone presence/*n* (%)	14 (18.4%)	7 (18.4%)	1.000
Cytogenetics			
Diploids/*n* (%)	72 (94.7%)	35 (92.1%)	0.684
Others/*n* (%)	4 (5.3%)	3 (7.9%)	

*ALT* alanine transaminase, *ATG* anti-thymocyte globulin, *ANC* absolute neutrophil count, *Cr* creatinine, *CsA* cyclosporine A, *EPAG* eltrombopag, *NSAA* non-severe aplastic anaemia, *PNH* paroxysmal nocturnal haemoglobinuria, *SAA* severe aplastic anaemia.

### Clinical efficacy

**Fig. 1:**
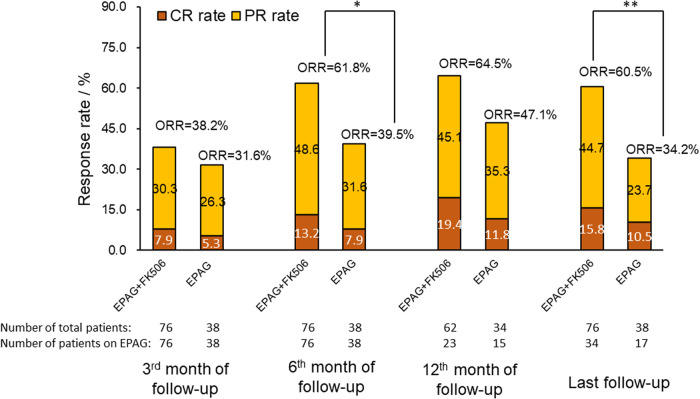
The response rates of patients in the EPAG+tacrolimus group and EPAG monotherapy group. The ORR to EPAG+tacrolimus was significantly higher than that to EPAG monotherapy at the 6^th^ month/last follow-up. CR complete response, EPAG eltrombopag, PR partial response, ORR overall response. *P < 0.05, **P < 0.01.

The ORR for patients treated with EPAG+tacrolimus and EPAG alone was 38.2% *vs*. 31.6% (*P* = 0.490) at the 3^rd^ month, 61.8% *vs*. 39.5% (*P* = 0.024) at the 6^th^ month, 64.5% *vs*. 47.1% (*P* = 0.097) at the 12^th^ month, and 60.5% *vs*. 34.2% (*P* = 0.008) at the last follow-up, with a median follow-up of 18 (6–24) months, comparable length of EPAG treatment (12 (6–24) months *vs*. 10 (6–24) months, *P* = 0.434), EPAG dose at the 3^rd^ month of follow-up (100 (25–150) mg/d *vs*. 100 (50–150) mg/d, *P* = 0.552), EPAG dose at the 6^th^ month of follow-up (75 (25–150) mg/d *vs*.100 (50–150) mg/d, *P* = 0.161), and the aggregated doses of EPAG exposure (26.5 (11.9–56.9)g *vs*. 27.0 (13.5–56.9)g, *P* = 0.559) for the EPAG+tacrolimus and EPAG monotherapy groups. No significant difference was found in the time to response (3 (1–12) months *vs*. 3 (1–8) months, *P* = 0.763) or the CRR at the 3^rd^ month/6^th^ month/12^th^ month/last follow-up between two groups (7.9%/13.2%/19.4%/15.8% *vs*. 5.3%/7.9%/11.8%/10.5%, *P* > 0.05 at each evaluated time point, Fig. 1).

### Safety

AEs of various degrees attributed to either EPAG or tacrolimus were reported in 23 (30.3%) patients with EPAG+tacrolimus and 9 (23.4%) patients with EPAG only. The most common AEs in the EPAG+tacrolimus group included dyspepsia (11.8%), increased creatinine (3.9%), and pruritus (3.9%); the most common AEs in the EPAG monotherapy group included dyspepsia (10.6%), elevated ALT (5.3%) and pruritus (2.6%). All AEs were CTCAE grade 1-2 and were relieved after symptomatic treatment or EPAG dose adjustment. No patient discontinued EPAG or died due to AEs. There was no significant difference in the rate of different AEs between the EPAG+tacrolimus group and the EPAG monotherapy group (Table 2).

**Table 2:** Adverse events in EPAG+tacrolimus and EPAG monotherapy group.

Adverse events	EPAG+tacrolimus *N* = 76	EPAG *N* = 38	*P-*value
Dyspepsia, *n* (%)			
Grade 2	2 (2.6%)	2 (5.3%)	0.600
Grade 1	7 (9.2%)	2 (5.3%)	0.715
Skin pruritus, *n* (%)			
Grade 2	1 (1.3%)	0 (0.0%)	1.000
Grade 1	2 (2.6%)	1 (2.6%)	1.000
Arthralgia, *n* (%)			
Grade 2	1 (1.3%)	0 (0.0%)	1.000
Grade 1	2 (2.6%)	1 (2.6%)	1.000
Elevated Cr, *n* (%)			
Grade 2	0 (0.0%)	0 (0.0%)	1.000
Grade 1	3 (3.9%)	1 (2.6%)	1.000
Elevated ALT, *n* (%)			
Grade 2	0 (0.0%)	2 (5.3%)	0.109
Grade 1	0 (0.0%)	0 (0.0%)	1.000

*ALT* alanine transaminase, *Cr* creatinine, *EPAG* eltrombopag.

### Survival and clonal evolution

**Fig. 2:**
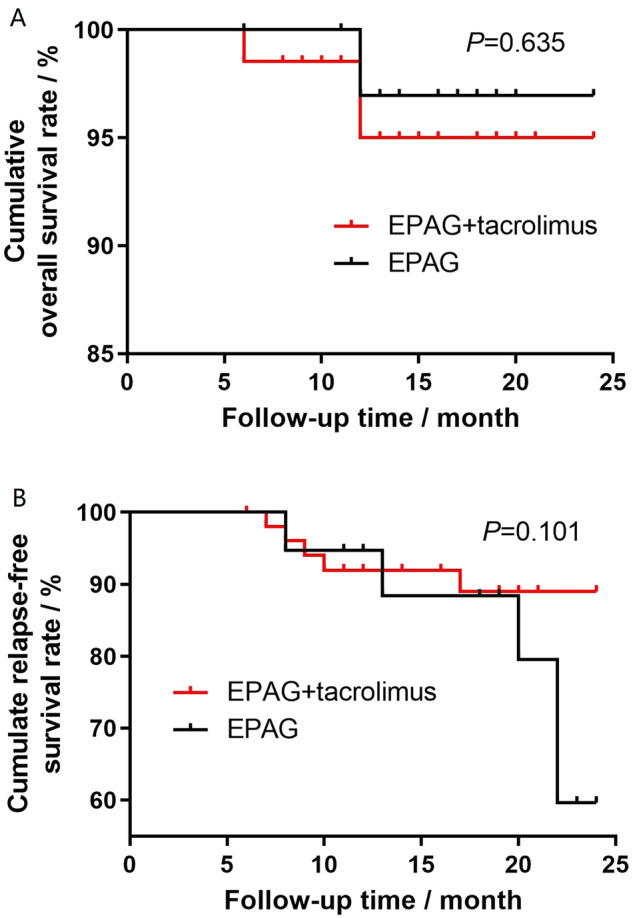
Cumulative overall survival (OS) and cumulative relapse-free survival (RFS) curves of the EPAG+tacrolimus and EPAG monotherapy groups. **A** The OS curve was not significantly different between the groups (*P* = 0.635). **B** The RFS curve showed a trend of benefit in the EPAG+tacrolimus group. EPAG eltrombopag.

Three patients (3.9%) died in the EPAG+tacrolimus group during follow-up, and the causes of death included pulmonary infection related to neutropenia for two patients and unknown reasons for one patient. None of the three patients responded, and they died at a median of 5 (1–6) months after discontinuing treatment. On the other hand, one NR patient (2.6%) in the EPAG monotherapy group died from unknown reasons at 3 months after discontinuing EPAG. No significant difference was found in the cumulative overall survival curve between the groups (*P* = 0.635, Fig. 2A).

Malignant clonal evolution occurred in 3 (3.9%) patients in the EPAG+tacrolimus group and one (2.6%) patient in the EPAG monotherapy group (*P* = 1.000, Supplementary Table 1). For the EPAG+tacrolimus group, one NSAA patient had monosomy 7 at the 12^th^ month, one SAA patient had a low-risk evolution event (trisomy 8) at the 13^th^ month but did not progress to MDS, and one SAA patient progressed to MDS (MDS with low blasts, according to WHO 2022 classification) at the 13^th^ month of follow-up. One SAA patient in the EPAG monotherapy group had t [10, 11] (p12; q21) at the 24^th^ month of follow-up but did not progress to MDS. An increase in PNH clone size occurred in 3 (3.9%) other patients in the EPAG+tacrolimus group at the 5th or 6th month of follow-up: one patient’s PNH clone size increased from 5% to 33%, one from 0% to 4%, and one from 0% to 3%. For the EPAG monotherapy group, one patient’s PNH clone size increased from 0% to 3% at the 9^th^ month of follow-up. All 8 patients mentioned above responded before clonal evolution events occurred.

### Predictors of overall response and relapse

Possible predictors of the ORR and relapse rate (RR) were analysed. For the EPAG+tacrolimus group, responders had a significantly higher baseline reticulocyte count (61.5 (11.5–230.5) × 10^9^/L *vs*. 21.6 (6.6–56.6) × 10^9^/L, *P* = 0.039) than nonresponders, but no other significant predictors were detected. Multivariate analysis showed that no factor was independently related to the ORR in the EPAG+tacrolimus group. For the EPAG monotherapy group, no significant predictor was found for the ORR (Table 3). No significant predictor was found for relapse in either the EPAG+tacrolimus or the EPAG monotherapy group (Supplementary Table 2).

**Table 3:** Factors that possibly affected the ORR of patients in EPAG+tacrolimus group and EPAG monotherapy group.

	EPAG+tacrolimus			EPAG monotherapy		
Patient characteristics	OR patients (*N* = 51)	NR patients (*N* = 25)	*P-*value	OR patients (*N* = 19)	NR patients (*N* = 19)	*P-*value
Male/n (%)	17 (33.3%)	10 (40.0%)	0.568	12 (63.2%)	7 (36.8%)	0.105
Age at EPAG initiation/years, median (range)	45 (15–74)	46 (14–83)	0.899	48 (18–79)	40 (14–78)	0.271
Patients ≥ 60 years old/*n* (%)	10 (19.6%)	6 (24.0%)	0.659	7 (36.8%)	6 (31.6%)	1.000
Interval between diagnosis and EPAG initiation/months, median (range)	24 (6–480)	36 (6–360)	0.798	96 (6–468)	53 (6–204)	0.390
Refractory/*n* (%)	27 (52.9%)	13 (52.0%)	0.938	12 (63.2%)	7 (36.8%)	0.105
NSAA/*n* (%)	42 (82.4%)	23 (92.0%)	0.321	18 (94.7%)	13 (68.4%)	0.090
Platelet count/×10^9^/L, median (range)	13 (1–40)	12 (1–261)	0.430	9 (1–26)	17 (2–26)	0.584
Absolute neutrophil count/×10^9^/L, median (range)	1.51 (0.18–7.45)	1.20 (0.36–4.68)	0.366	1.76 (0.61–4.45)	1.02 (0.80–3.3)	0.238
Haemoglobin / (g/L), median (range)	82 (24–159)	81 (44–137)	0.866	77 (30–167)	63 (36–126)	0.242
Reticulocyte count/×10^9^/L, median (range)	61.5 (11.5–230.5)	21.6 (6.6–56.6)	**0.039**	51.9 (16.6–220.6)	50.1 (16.6–54.1)	0.535
ALT/(U/L), median (range)	17 (6–118)	13 (5–101)	0.591	18 (11–32)	17 (8–32)	0.645
Cr/(μmol/mL), median (range)	80 (38–118)	83 (56–168)	0.558	93 (40–178)	79 (43–178)	0.437
Ferritin/(ng/mL), median (range)	466 (12–4324)	523 (10–4420)	0.282	762 (12–9135)	1530 (270–2567)	0.285
Cytogenetics mutation presence/*n* (%)	2 (3.9%)	2 (8.0%)	0.594	2 (10.5%)	1 (5.2%)	1.000
PNH clone presence at EPAG initiation/*n* (%)	9 (17.6%)	5 (20.0%)	1.000	3 (15.8%)	4 (21.1%)	1.000

*ALT* alanine transaminase, *Cr* creatinine, *EPAG* eltrombopag, *NR* no response, *NSAA* non-severe aplastic anaemia, *OR* overall response, *PNH* paroxysmal nocturnal haemoglobinuria.

Bold values identify statistical significance (*p* < 0.05).

### Post hoc analysis of RFS and age subgroups

**Fig. 3:**
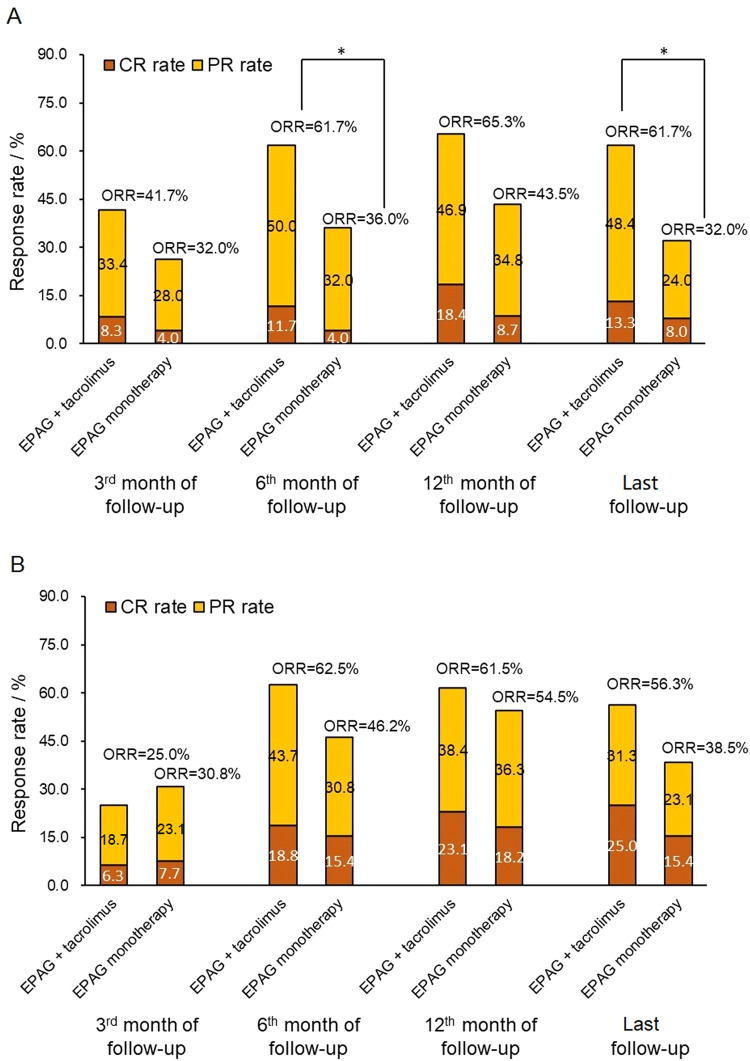
The response rate of patients in different age subgroups. **A** The response rate of patients <60 years old revealed a significantly higher ORR at the 6^th^ month/last follow-up in patients treated with EPAG+tacrolimus than in those treated with EPAG alone. **B** The response rate of patients ≥60 years old was not significantly different between patients treated with either EPAG+tacrolimus or EPAG monotherapy at any evaluated time point. CR complete response, EPAG eltrombopag, PR partial response, ORR overall response. *P < 0.05.

**Fig. 4:**
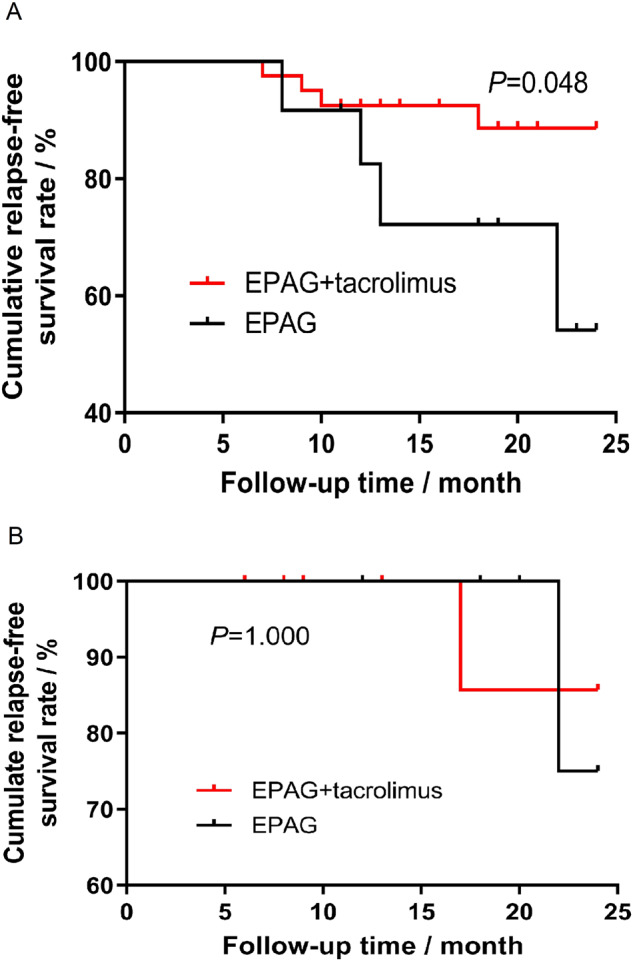
Cumulative relapse-free survival (RFS) of patients of different ages. **A** Cumulative RFS curves of patients <60 years old illustrated a significant benefit in the EPAG+tacrolimus group (*P* = 0.048). **B** Cumulative RFS curves of patients ≥60 years old illustrated no significant difference between the treatment groups. EPAG eltrombopag.

A post hoc analysis of RFS was conducted, and no difference was detected between the RFS curves of the two groups (*P* = 0.101, Fig. 2B). We also conducted a post hoc analysis to compare the ORR, CRR, RFS and clonal evolution in different age subgroups (<60 years old or ≥60 years old). A total of 78.9% (60/76) of patients receiving EPAG+tacrolimus and 65.8% (25/38) of patients receiving EPAG monotherapy were <60 years old (*P* = 0.128). For patients under 60 years old, the baseline characteristics before EPAG were comparable (Supplementary Table 3). The ORR at the 6th month/last follow-up was significantly higher in patients with EPAG+tacrolimus than in those with EPAG monotherapy (61.7% *vs*. 36.0%, *P* = 0.030 at the 6^th^ month and 61.7% *vs*. 32.0%, *P* = 0.013 at the last follow-up, respectively), and for the ORR at the 3^rd^ month and 12^th^ month, the difference between the groups was not significant (Fig. 3A). The CRRs were 8.3%/11.7%/18.4%/13.3% at the 3rd month/6th month/12th month/last follow-up in the EPAG+tacrolimus group and 4.0%/4.0%/8.7%/8.0% in the EPAG monotherapy group (*P* > 0.05 for the CRR at each evaluated time point between the groups, Fig. 3A). In this subgroup, a clear benefit in RFS was observed in the EPAG+tacrolimus group (*P* = 0.048, Fig. 4A). There was no significant difference in the death rate (1.7% *vs*. 0.0%, *P* = 1.000) or malignant clonal evolution rate (3.3% *vs*. 4.0%, *P* = 1.000) between the therapy groups in this age subgroup.

For patients ≥60 years old, the baseline characteristics were comparable except for the proportion of males (18.8% *vs*. 61.5%, *P* = 0.027, Supplementary Table 4). No significant difference was found in the ORR (25.0%/62.5%/61.5%/56.3% *vs*. 30.8%/46.2%/54.5%/38.5%, *P* > 0.05, Fig. 3B) or CRR (6.3%/18.8%/23.1%/25.0% *vs*. 7.7%/15.4%/18.2%/15.4%, *P* > 0.05, Fig. 3B) at the 3^rd^/6^th^/12^th^/last follow-up between elderly patients with EPAG+tacrolimus and EPAG. No difference in cumulative RFS curves (*P* = 1.000, Fig. 4B), the death rate (12.5% *vs*. 7.7%, *P* = 1.000) or malignant clonal evolution rate (6.3% *vs*. 0.0%, *P* = 1.000) was found between the groups.

### Post hoc analysis of the relapsed/refractory subgroups

We further conducted a post hoc subgroup analysis on relapsed and refractory patients. For the relapsed subgroup, there were 55 patients; 36 were in the EPAG+tacrolimus group, and 19 were in the EPAG monotherapy group. No significant differences were detected in the baseline characteristics between patients in the EPAG+tacrolimus and EPAG monotherapy groups except for in the baseline haemoglobin level (88 (44–159) g/L for the EPAG+tacrolimus group and 68 (30-114) g/L for the EPAG monotherapy group, *P* = 0.032, Supplementary Table 5). The ORR was significantly higher in the 6th month of follow-up and the last follow-up (58.3% *vs*. 26.3% in the 6^th^ month of follow-up, *P* = 0.024; 58.3% *vs*. 26.3% at the last follow-up, *P* = 0.024), while no significant difference was found in the ORR at the 3rd/12th month of follow-up (Supplementary Fig. 2A). The CRR was 11.1%/13.9%/10.7%/13.9% and 5.3%/5.3%/5.6%/5.3% (*P* > 0.05) for the EPAG+tacrolimus group and EPAG monotherapy group at the 3^rd^ month/6^th^ month/12^th^ month/last follow-up, respectively. No significant difference in the cumulative RFS curves was found between relapsed patients in the EPAG+tacrolimus and EPAG monotherapy groups (*P* = 0.422, Supplementary Fig. 3A). The death rates were 2.8% and 0% (*P* = 1.000) for relapsed patients in the EPAG+tacrolimus and EPAG monotherapy groups, respectively.

There were 59 refractory patients, with 40 patients in the EPAG+tacrolimus group and 19 in the EPAG monotherapy group. No significant difference was found in baseline characteristics (Supplementary Table 6) for refractory patients in the EPAG+tacrolimus and EPAG monotherapy groups. No significant difference was found in the ORR and CRR at the 3rd month/6th month/12th month/last follow-up (Supplementary Fig. 2B). No significant difference in the cumulative RFS curves was found between refractory patients in the EPAG+tacrolimus and EPAG monotherapy groups, yet a trend of superiority in the cumulative RFS of the EPAG+tacrolimus group was observed (*P* = 0.106, Supplementary Fig. 3B). The death rates were 5.0% and 5.3% (*P* = 1.000) for relapsed patients in the EPAG+tacrolimus and EPAG monotherapy groups, respectively.

## Discussions

As a TPO-RA, EPAG has been found to enhance haematopoiesis in AA patients and has recently been applied in AA treatment. Common EPAG application in AA includes EPAG monotherapy and EPAG combination therapy, among which EPAG + ATG+CsA or EPAG plus oral immunosuppressive therapy is the major option. A phase-3 randomized clinical trial found that EPAG + ATG+CsA was superior in terms of the ORR, CRR and duration of response compared with ATG+CsA for newly diagnosed SAA patients [13]. However, few studies have focused on the efficacy and safety of EPAG plus immunosuppressive agents in patients with refractory/relapsed AA. Gao et al. reported their findings of a retrospective cohort study of EPAG combined with oral immunosuppressants (CsA or tacrolimus) and androgen therapy in patients with refractory/relapsed AA. The ORR of the patients in this cohort was 42% [16], but the number of included patients was very limited. Some other studies included relapsed/refractory AA patients treated with EPAG with or without oral immunosuppressants but did not report the efficacy of each different EPAG therapy regimen [17, 18] and failed to compare the efficacy and safety between EPAG+immunosuppressive therapy and EPAG monotherapy directly. In addition, these studies were all retrospective studies. The current trial was the first large randomized prospective trial to compare the efficacy and safety of EPAG+tacrolimus and EPAG monotherapy for refractory/relapsed AA patients and their long-term survival and to analyse the factors that possibly affected the response rate or relapse rate associated with each therapy.

For our primary outcome, we found that for refractory/relapsed AA patients, EPAG+tacrolimus was superior in terms of the ORR at the 6th month compared with EPAG monotherapy. Like CsA, Tacrolimus is also an inhibitor of calcineurin. Compared with CsA, tacrolimus has 100 times stronger inhibitory activity on calcineurin, no side effects of hair hyperplasia and gingival hyperplasia and less damage to renal function. Alsultan et al. [19] and Zhu et al. [20] reported similar efficiency of ATG+tacrolimus and ATG+CsA for children SAA and adult SAA patients. Du et al. reported that the conversion to tacrolimus induced an ORR of 38.6% for 101 NSAA patients who were nonresponders to or intolerant of CsA [15]. The findings indicated that tacrolimus could be an alternative for CsA in terms of combination with EPAG, and that the addition of tacrolimus to EPAG could further improve the efficacy of EPAG monotherapy. In the present study, there was a significant difference in the ORR at the 6th month and the last follow-up. There was no significant difference in the ORR between the therapy groups at the 12^th^ month of follow-up, but the difference seemed to be close to significant. In addition, the CRR for EPAG+tacrolimus was numerically higher than that for EPAG monotherapy, although the difference was not significant. In general, our findings suggested that adding tacrolimus to EPAG could improve treatment efficacy in AA patients to some extent compared with EPAG alone. EPAG + IST has been reported to show advantage towards IST alone in untreated SAA patients [13], while according to Patel et al., this advantage did not seem prominent for the SAA patients who relapsed after EPAG + IST [21]. In Patel et al.’s study, patients who relapsed after EPAG + IST received either EPAG+CsA or CsA treatment. Blood counts recovered in 93% of patients treated with CSA alone and in 60% of patients treated with CsA and EPAG. However, in Patel et al.’s study, no details were given about the condition of relapsed patients before CsA or CsA+EPAG treatment [21], so there might be some confounding factors for the efficacy of CsA and CsA+EPAG for relapsed AA patients in this study that were not discussed.

EPAG+tacrolimus was as tolerable as EPAG monotherapy in our trial. The AE rate of EPAG+tacrolimus in patients with refractory/relapsed AA was 20% to 30%, which was lower than the AE rate reported in previous studies on EPAG monotherapy for relapsed/refractory AA patients [17, 22]. The most common AEs in the EPAG+tacrolimus group and EPAG monotherapy group included dyspepsia and skin pruritus, which were generally consistent with the AEs reported in other studies [10, 16], and no severe AEs of CTCAE grades 3-4 were observed.

In this study, there was no significant difference in the death rate or overall survival curves between the EPAG+tacrolimus group and the EPAG monotherapy group. In previous studies on refractory/relapsed AA patients receiving EPAG therapy, the reported clonal evolution rate ranged between 8.0% and 14.3%, and the death rate fluctuated between 0% and 8% [11, 17]. In our study, adding tacrolimus to EPAG treatment did not increase/decrease the clonal evolution rate or death rate. All the patients who died during follow-up were NR patients, and in previous studies, failing to respond to EPAG treatment was associated with a lower survival rate [18].

Our study also analysed the predictors that possibly affected the response/relapse rate for EPAG+tacrolimus or EPAG monotherapy. For the EPAG+tacrolimus group, OR patients had significantly higher absolute reticulocyte counts than NR patients, although multivariate analysis found that the absolute reticulocyte count was not independently related to the ORR in the EPAG+tacrolimus group. The correlation between the absolute reticulocyte count and the ORR was also reported in a study on EPAG + ATG+CsA for newly diagnosed SAA patients [23]. For the EPAG monotherapy group, no influencing factors of the response rate were found, which was consistent with Ruan et al’s study [10]. Our study did not detect predictors for relapse events in either therapy group, which could be explained by the limited number of relapsed patients, which increased the difficulty of detecting significant differences.

The post hoc age subgroup analysis suggested that the superiority of EPAG+tacrolimus over EPAG monotherapy might be more prominent in the <60-year-old subgroup: the ORRs at the 6th month and the last follow-up were significantly higher, and the difference between RFS curves in this subgroup was also significant. The ORR of EPAG monotherapy group at 12^th^ month seemed to be higher and thus reduce the disparity in efficacy, which may be related to the bias caused by smaller number of analysed patients. The difference in the cumulative RFS between the EPAG+tacrolimus and EPAG monotherapy groups was also significant. These benefits were not observed in the ≥60-year-old subgroup. The above findings indicated that the superior clinical efficacy of EPAG+tacrolimus was mainly found in younger patients who were <60 years old, rather than in older patients. For older patients, the tolerance for either tacrolimus or EPAG might decrease; furthermore, previous studies also showed that the prominent advantage of EPAG + ATG+CsA was mainly found in younger adult AA patients [13]. Even for IST alone, the response was better for younger patients. Bacigalupo et al. compared the treatment efficacy for AA patients <20 years, 21–40 years, 41–60 years and >60 years old who were treated with IST and found that the efficacy of IST increased when patients were younger [24]. Nevertheless, it is worth mentioning that for untreated AA patients <18 years old, adding EPAG to standard IST did not improve the efficacy compared with IST alone [25], so our findings might not be applicable in the paediatric population.

A post hoc analysis of relapsed and refractory patients separately revealed that the advantage of EPAG+tacrolimus over EPAG monotherapy seemed to be more obvious in relapsed patients than in refractory patients. The baseline haemoglobin level of relapsed patients receiving EPAG+tacrolimus treatment was higher than that of patients receiving EPAG monotherapy, but baseline haemoglobin has not been identified as a factor related to response in previous studies on EPAG + IST or EPAG treatment for AA patients. This finding was reasonable since refractory patients had failed to respond to IST before they were enrolled, and the addition of tacrolimus, another immunosuppressant, might not be helpful for this subgroup of patients.

This is a large study of EPAG treatment for refractory/relapsed AA patients, and to our knowledge, it is the first trial to compare the efficacy and safety of EPAG+tacrolimus and EPAG monotherapy in aplastic anaemia patients. The present study found that the clinical efficacy of EPAG+tacrolimus was better than that of EPAG monotherapy, and the safety and clonal evolution rate of the two groups were comparable. In addition, post hoc analysis indicated that the superiority of EPAG+tacrolimus tended to be more prominent in younger and relapsed patients. Nevertheless, further studies are needed to compare tacrolimus to CsA to figure out whether tacrolimus could become a superior alternative for CsA in terms of combination with EPAG. There are some limitations in our study: to guarantee a better tolerance of EPAG for enrolled patients, a 2-week ramp-up of EPAG to a maximum dose was set, and this regimen possibly contributed to the lack of a difference in patient response observed at the 3^rd^ month or even a lower response rate in both arms during follow-up. In addition, it needs to be clarified that the majority of our enrolled patients were NSAA patients; therefore, our findings may not be generalizable to the SAA population directly, and further related trials on SAA patients may be essential. Nevertheless, the findings above have implications for the application of EPAG+tacrolimus in patients with refractory/relapsed AA.

### Supplementary information

Supplementary Materials

## Data Availability

The datasets generated during and analysed during the current study are available from the corresponding authors on reasonable request.
