# Supplementary material for: Eltrombopag with or without Tacrolimus for relapsed/refractory acquired aplastic anaemia: a prospective randomized trial
Source: Blood Cancer J. 2023 Sep 19;13(1):146. doi: 10.1038/s41408-023-00921-8 (PMC10509202; doi:10.1038/s41408-023-00921-8)
Supplement: Supplementary file 1 — Supplementary Materials [file 41408_2023_921_MOESM1_ESM.docx]

**Supplementary Table 1** Malignant clone evolution events of patients

| Patient age / sex | Therapy received | Length of therapy at evolution (month) | Cytogenetics | Somatic mutations | Bone marrow blast | Patient outcome |
| --- | --- | --- | --- | --- | --- | --- |
| 55/M | EPAG + tacrolimus | 13 | None | *BCOR, DNMT3A, RB1, GNAS* | 1% | Stopped EPAG |
| 45/M | EPAG + tacrolimus | 13 | Trisomy 8 | None | 0 | Tapered EPAG and did not relapse |
| 67/F | EPAG + tacrolimus | 12 | Monosomy 7 | None | 0 | Tapered EPAG and did not relapse |
| 46/M | EPAG monotherapy | 24 | t (10,11) (p12; q21) | None | 0 | Followed up as scheduled |

EPAG, eltrombopag; F, female; M, male

**Supplementary Table 2** Factors that possibly affected relapse rate of responded patients in EPAG+tacrolimus group and EPAG monotherapy group

|  | EPAG+tacrolimus | | | EPAG monotherapy | | |
| --- | --- | --- | --- | --- | --- | --- |
| Patient characteristics | **Relapsed patients**  **(N=5)** | **Non-relapsed patients**  **(N=46)** | ***P* value** | **Relapsed patients**  **(N=5)** | **Non-relapsed patients**  **(N=14)** | ***P* value** |
| Male / n (%) | 2 (40.0%) | 15 (32.6%) | 1.000 | 4 (80.0%) | 8 (57.1%) | 1.000 |
| Age at EPAG initiation / years, median (range) | 40 (18-67) | 47 (15-74) | 0.527 | 46 (33-69) | 48 (18-79) | 0.754 |
| Interval between diagnosis and EPAG initiation / months, median (range) | 120 (6-228) | 24 (6-480) | 0.431 | 96 (9-216) | 82.5 (6-468) | 0.559 |
| Refractory / n (%) | 2 (40.0%) | 25 (54.3%) | 0.656 | 3 (60.0%) | 9 (64.3%) | 1.000 |
| NSAA / n (%) | 3 (60.0%) | 39 (84.8%) | 0.209 | 5 (100.0%) | 13 (92.9%) | 1.000 |
| Platelet count / ×10^9^/L, median (range) | 16 (3-29) | 13 (1-40) | 0.765 | 16 (9-26) | 7.5 (1-25) | 0.186 |
| Absolute neutrophil count / ×10^9^/L, median (range) | 1.26 (0.98-3.44) | 1.52 (0.18-7.45) | 0.906 | 1.08 (0.66-3.80) | 1.95 (0.61-4.45) | 0.800 |
| Haemoglobin / (g/L), median (range) | 78 (50-110) | 82 (24-159) | 0.602 | 66 (47-146) | 88.5 (30-167) | 0.687 |
| Reticulocyte count / ×10^9^/L,  median (range) | 51.05  (13.2-88.9) | 61.5  (11.5-230.5) | 0.758 | 48.0  (44.2-51.9) | 56.2  (16.6-220.6) | 0.857 |
| ALT / (U/L), median (range) | 11 (9-118) | 17 (6-72) | 0.883 | 16 (14-18) | 19 (11-32) | 0.513 |
| Cr / (μmol/mL), median (range) | 53 (53-88) | 80 (38-118) | 0.192 | 110 (66-178) | 92 (40-178) | 0.291 |
| Ferritin / (ng/mL), median (range) | 767 (13-4064) | 466 (12-4324) | 0.984 | 1996 (108-2876) | 232 (12-9135) | 0.343 |
| Cytogenetics mutation presence  / n (%) | 0 (0.0%) | 2 (4.3%) | 1.000 | 1 (20.0%) | 1 (7.1%) | 0.468 |
| PNH clone presence at EPAG initiation / n (%) | 2 (40.0%) | 7 (15.2%) | 0.209 | 0 (0.0%) | 3 (21.4%) | 0.530 |

ALT, alanine transaminase; Cr: creatinine; EPAG, eltrombopag; NR, no response; NSAA, non-severe aplastic anaemia; OR, overall response; PNH, paroxysmal nocturnal haemoglobinuria

**Supplementary Table 3** Baseline characteristics of patients < 60 years old

| Characteristics | EPAG+tacrolimus  N=60 | EPAG  N=25 | *P* value |
| --- | --- | --- | --- |
| Age at EPAG initiation / years, median (rage) | 39 (14-57) | 38 (14-56) | 0.696 |
| Male / n (%) | 24 (40.0%) | 11 (44.0%) | 0.733 |
| Severity of AA at EPAG initiation | | |  |
| NSAA / n (%) | 53 (88.3%) | 21 (84.0%) | 0.724 |
| SAA / n (%) | 7 (11.7%) | 4 (16.0%) |  |
| Disease status |  | |  |
| Relapsed / n (%) | 30 (50.0%) | 15 (60.0%) | 0.400 |
| Refractory / n (%) | 30 (50.0%) | 10 (40.0%) |  |
| Previous treatment |  | |  |
| ATG+CsA / n (%) | 7 (11.7%) | 4 (16.0%) | 0.724 |
| CsA / n (%) | 53 (88.3%) | 21 (84.0%) |  |
| Time from diagnosis to EPAG initiation / months, median (range) | 36.5 (6-384) | 60 (6-468) | 0.335 |
| ANC / ×10^9^/L, median (range) | 1.52 (0.33-7.45) | 1.07 (0.61-4.45) | 0.872 |
| Platelet count / ×10^9^/L, median (range) | 14 (1-45) | 16 (1-26) | 0.390 |
| Haemoglobin / (g/L), median (range) | 86 (24-159) | 77 (30-167) | 0.441 |
| Reticulocyte count / ×10^9^/L, | 58.0 (6.6-230.5) | 51.9 (16.6-220.6) | 0.808 |
| median (range) |  |  |  |
| Cr / (μmol/mL), median (range) | 83 (38-168) | 79 (40-110) | 0.583 |
| ALT / U/L, median (range) | 14 (5-101) | 20 (8-32) | 0.354 |
| Ferritin / ng/ml, median (range) | 459 (10-4420) | 960 (12-9135) | 0.598 |
| PNH clone presence / n (%) | 11 (18.3%) | 4 (16.0%) | 1.000 |
| Cytogenetics |  |  |  |
| Diploids / n (%) | 57 (95.0%) | 23 (92.0%) | 0.628 |
| Others / n (%) | 3 (5.0%) | 2 (8.0%) |  |

ALT, alanine transaminase; ATG, anti-thymocyte globulin; ANC, absolute neutrophil count; Cr, creatinine; EPAG, eltrombopag; NR, no response; NSAA, non-severe aplastic anaemia; OR, overall response; PNH, paroxysmal nocturnal haemoglobinuria; SAA, severe aplastic anaemia

**Supplementary Table 4** Baseline characteristics of patients ≥ 60 years old

| Characteristics | EPAG+tacrolimus | EPAG | *P* value |
| --- | --- | --- | --- |
|  | **N=16** | **N=13** |  |
| Age at EPAG initiation / years, median (rage) | 66 (60-83) | 69 (64-79) | 0.092 |
| Male / n (%) | 3 (18.8%) | 8 (61.5%) | **0.027** |
| Severity of AA at EPAG initiation | | |  |
| NSAA / n (%) | 12 (75.0%) | 10 (76.9%) | 1.000 |
| SAA / n (%) | 4 (25.0%) | 3（23.1%) |  |
| Disease status |  | |  |
| Relapsed / n (%) | 6 (37.5%) | 4 (30.8%) | 1.000 |
| Refractory / n (%) | 10 (62.5%) | 9 (69.2%) |  |
| Previous treatment |  | |  |
| ATG+CsA / n (%) | 4 (25.0%) | 3（23.1%) | 1.000 |
| CsA / n (%) | 12 (75.0%) | 10 (76.9%) |  |
| Time from diagnosis to EPAG initiation / months, median (range) | 20.5 (6-480) | 65 (6-340) | 0.329 |
| ANC / ×10^9^/L, median (range) | 1.32 (0.18-4.13) | 1.45 (0.66-2.77) | 0.961 |
| Platelet count / ×10^9^/L, median (range) | 6.5 (1-261) | 14 (3-24) | 0.786 |
| Haemoglobin / (g/L), median (range) | 66 (45-148) | 62 (37-136) | 0.248 |
| Reticulocyte count / ×10^9^/L, | 35.0 (13.2-65.6) | 44.2 (16.6-62.9) | 0.836 |
| median (range) |  |  |  |
| Cr / (μmol/mL), median (range) | 75.5 (53-117) | 93 (48-178) | 0.569 |
| ALT / U/L, median (range) | 16 (6-118) | 14 (10-23) | 0.766 |
| Ferritin / ng/ml, median (range) | 1231 (32-2784) | 1873 (32-4018) | 0.161 |
| PNH clone presence / n (%) | 3 (18.8%) | 3 (23.1%) | 1.000 |
| Cytogenetics |  |  |  |
| Diploids / n (%) | 15 (93.8%) | 12 (92.3%) | 1.000 |
| Others / n (%) | 1 (6.3%) | 1 (7.7%) |  |

ALT, alanine transaminase; ATG, anti-thymocyte globulin; ANC, absolute neutrophil count; Cr, creatinine; EPAG, eltrombopag; NR, no response; NSAA, non-severe aplastic anaemia; OR, overall response; PNH, paroxysmal nocturnal haemoglobinuria; SAA, severe aplastic anaemia

**Supplementary Table 5** Baseline characteristics of relapsed patients

| Characteristics | EPAG+tacrolimus | EPAG | *P* value | |  |
| --- | --- | --- | --- | --- | --- |
|  | **N=36** | **N=19** |  |  |  |
| Age at EPAG initiation / years, median (rage) | 40 (15-83) | 40 (14-79) | 0.936 | |  |
| Male / n (%) | 12 (33.3%) | 8 (42.1%) | 0.520 | |  |
| Severity of AA at EPAG initiation | | |  | |  |
| NSAA / n (%) | 33 (91.7%) | 16 (84.2%) | 0.405 | |  |
| SAA / n (%) | 3 (8.3%) | 3 (15.8%) |  |  |  |
| Previous treatment |  | |  | |  |
| ATG+CsA / n (%) | 3 (8.3%) | 3 (15.8%) | 0.405 | |  |
| CsA / n (%) | 33 (91.7%) | 16 (84.2%) |  |  |  |
| Time from diagnosis to EPAG initiation / months, median (range) | 68.5 (6-480) | 96 (20-468) | 0.288 | |  |
| ANC / ×10^9^/L, median (range) | 1.50 (0.33-7.19) | 1.02 (0.61-3.80) | 0.353 | |  |
| Platelet count / ×10^9^/L, median (range) | 17 (1-261) | 14 (1-26) | 0.099 | |  |
| Haemoglobin / (g/L), median (range) | 88 (44-159) | 68 (30-114) | 0.032 | |  |
| Reticulocyte count / ×10^9^/L, | 58.0 (6.6-134.0) | 50.1 (16.6-56.23) | | 0.197 | |
| median (range) |  |  |  |  |  |
| Cr / (μmol/mL), median (range) | 84 (38-168) | 72 (40-178) | 0.664 | |  |
| ALT / U/L, median (range) | 15.5 (5-45) | 14 (8-30) | 0.930 | |  |
| Ferritin / ng/ml, median (range) | 294 (10-4324) | 1061 (32-9135) | 0.307 | |  |
| PNH clone presence / n (%) | 5 (13.9%) | 3 (15.8%) | 1.000 | |  |
| Cytogenetics |  |  |  | |  |
| Diploids / n (%) | 33 (91.7%) | 16 (84.2%) | 0.405 | |  |
| Others / n (%) | 3 (8.3%) | 3 (15.8%) |  |  |  |

ALT, alanine transaminase; ATG, anti-thymocyte globulin; ANC, absolute neutrophil count; Cr, creatinine; EPAG, eltrombopag; NR, no response; NSAA, non-severe aplastic anaemia; OR, overall response; PNH, paroxysmal nocturnal haemoglobinuria; SAA, severe aplastic anaemia

**Supplementary Table 6** Baseline characteristics of refractory patients

| Characteristics | EPAG+tacrolimus | EPAG | *P* value |
| --- | --- | --- | --- |
|  | **N=40** | **N=19** |  |
| Age at EPAG initiation / years, median (rage) | 48 (14-79) | 54 (16-78) | 0.098 |
| Male / n (%) | 15 (37.5%) | 11 (57.9%) | 0.140 |
| Severity of AA at EPAG initiation | | |  |
| NSAA / n (%) | 32 (80.0%) | 15 (78.9%) | 1.000 |
| SAA / n (%) | 8 (20.0%) | 4（21.1%) |  |
| Previous treatment |  | |  |
| ATG+CsA / n (%) | 8 (20.0%) | 4（21.1%) | 1.000 |
| CsA / n (%) | 32 (80.0%) | 16 (78.9%) |  |
| Time from diagnosis to EPAG initiation / months, median (range) | 9 (6-384) | 30 (6-204) | 0.323 |
| ANC / ×10^9^/L, median (range) | 1.48 (0.18-7.45) | 1.57 (0.78-4.45) | 0.460 |
| Platelet count / ×10^9^/L, median (range) | 11.5 (1-45) | 19 (4-26) | 0.232 |
| Haemoglobin / (g/L), median (range) | 73 (24-148) | 70 (38-167) | 0.743 |
| Reticulocyte count / ×10^9^/L, | 45.2 (8.5-230.5) | 51.9 (16.6-220.6) | 0.650 |
| median (range) |  |  |  |
| Cr / (μmol/mL), median (range) | 79 (52-118) | 91.5 (64-178) | 0.155 |
| ALT / U/L, median (range) | 13.5 (6-118) | 20.5 (12-32) | 0.219 |
| Ferritin / ng/ml, median (range) | 831.5 (12-4420) | 1364 (12-4018) | 0.417 |
| PNH clone presence / n (%) | 9 (22.5%) | 2 (15.4%) | 0.711 |
| Cytogenetics |  |  |  |
| Diploids / n (%) | 39 (97.5%) | 13 (100%) | 1.000 |
| Others / n (%) | 1 (2.5%) | 0 (0%) |  |

ALT, alanine transaminase; ATG, anti-thymocyte globulin; ANC, absolute neutrophil count; Cr, creatinine; EPAG, eltrombopag; NR, no response; NSAA, non-severe aplastic anaemia; OR, overall response; PNH, paroxysmal nocturnal haemoglobinuria; SAA, severe aplastic anaemia


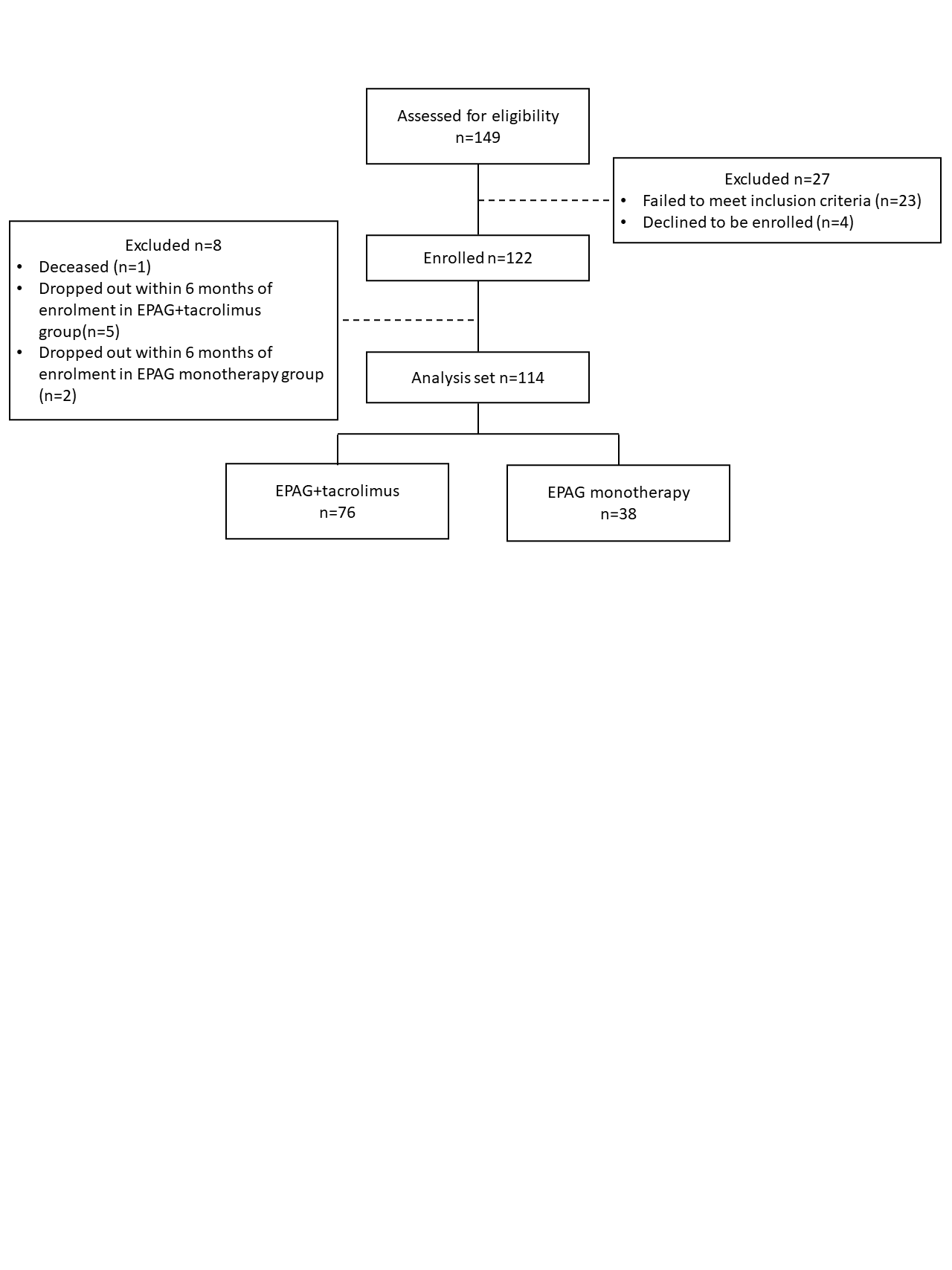


**Supplementary Figure 1** CONSORT diagram illustrating patient screening.


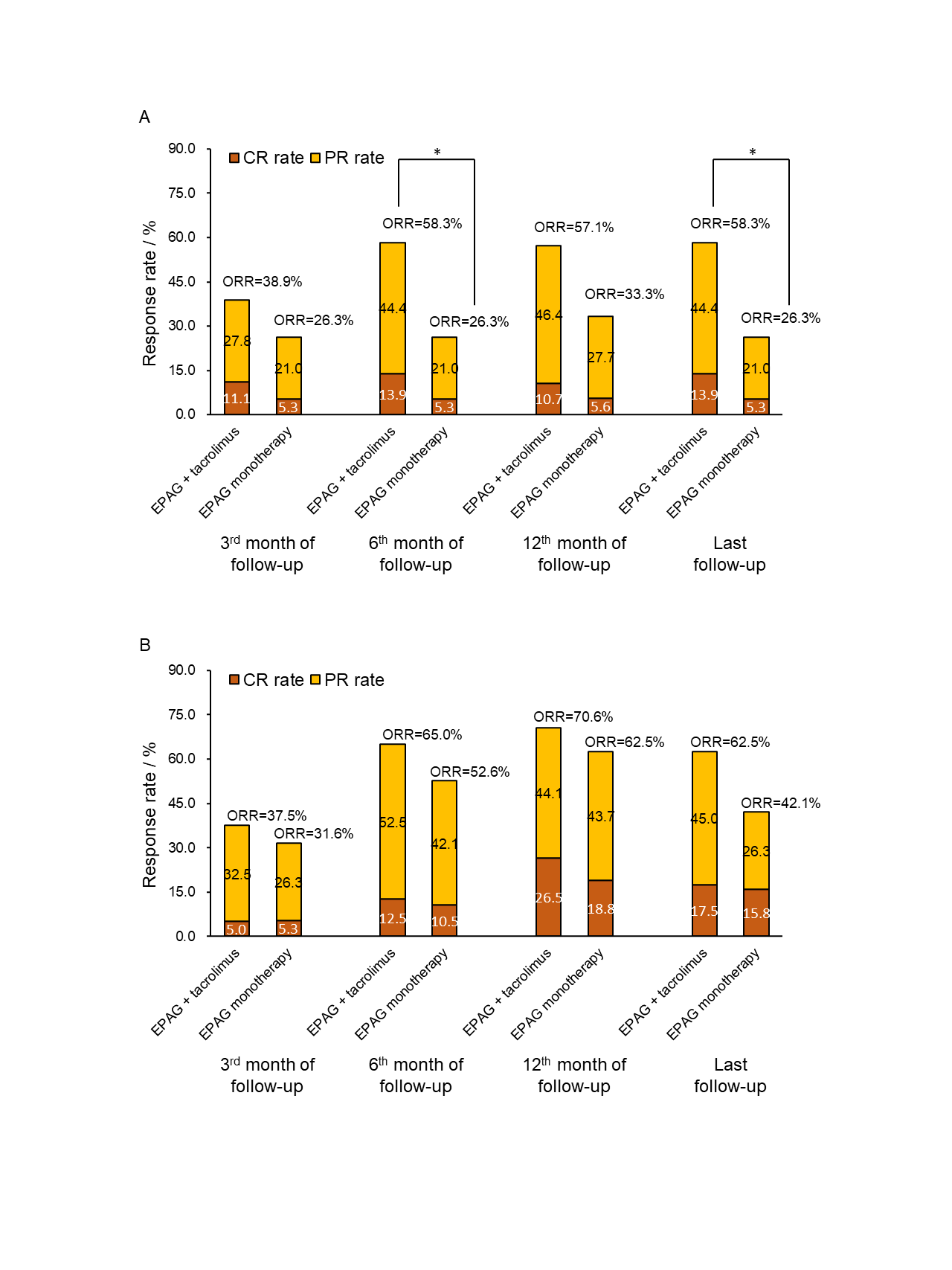


**Supplementary Figure 2** The response rate of patients in relapsed/refractory subgroups. (A) The response rate of relapsed patients revealed a significant higher ORR at the 6^th^ month / last follow-up in patients treated with EPAG+tacrolimus compared with those with EPAG alone. (B) The response rate of refractory patients showed no significant difference between patients treated with either EPAG+tacrolimus or EPAG monotherapy at any evaluated time point. CR, complete response; EPAG, eltrombopag; PR partial response; ORR, overall response. **P*<0.05.


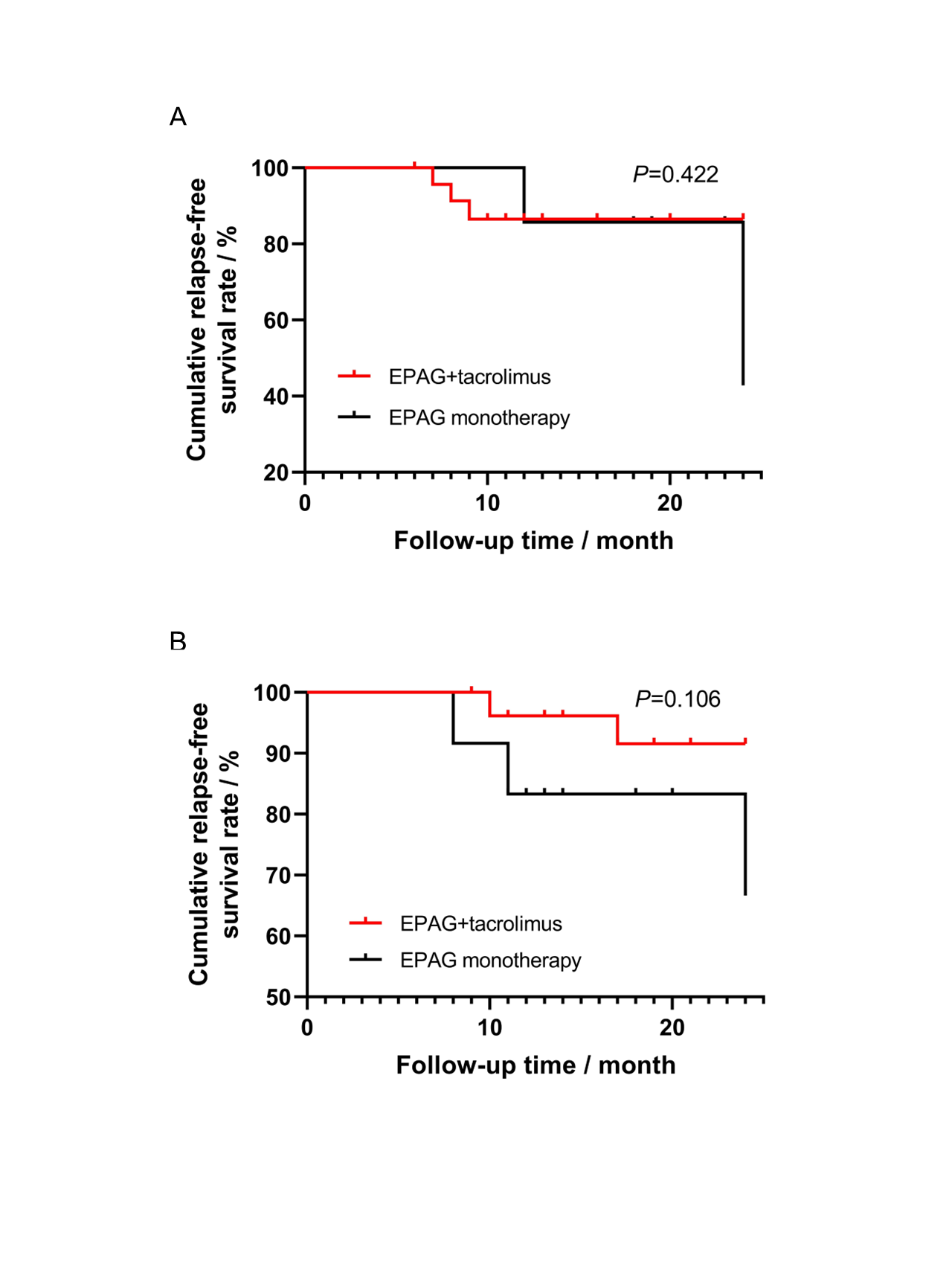


**Supplementary Figure 3** Cumulative relapse-free survival (RFS) of relapsed/refractory patients. Cumulative RFS curves of relapsed patients (A) and refractory patients (B) illustrated no significant difference between EPAG+tacrolimus group and EPAG monotherapy group. EPAG, eltrombopag.
